# Biotransformation of quercetin by *Bacillus subtilis* and anticancer activity evaluation: in vitro and in Silico

**DOI:** 10.1186/s13568-025-01860-2

**Published:** 2025-04-02

**Authors:** Salsabeel N. El Gendy, Amira K. Elmotayam, Reham Samir, Marwa I. Ezzat, Mahmoud T. Abo-Elfadl, Aly M. EL Sayed

**Affiliations:** 1 Department of Pharmacognosy, Faculty of Pharmacy, Cairo University, Kasr El-Ainy Street, Cairo, 11562 Egypt; 2 Department of Microbiology and Immunology, Faculty of Pharmacy, Cairo University, Kasr El-Ainy Street, Cairo, 11562 Egypt; 3 Biochemistry Department, Biotechnology Research Institute, National Research Centre, Dokki, Cairo 12622 Egypt; 4 Cancer Biology and Genetics Laboratory, Centre of Excellence for Advanced Sciences, National Research Centre, Cairo, 12622 Egypt

**Keywords:** Microbial biotransformation, Apigenin-7-*O*-pentoside, Anticancer activity, PI3K*δ*, Cathepsin B, Docking study

## Abstract

Biotransformation of quercetin by *Bacillus subtilis* ATCC 23,857 yielded a major metabolite; apigenin-7-*O*-pentoside (A7P) and 9 other phenolic metabolites. They were elucidated by LC-ESI-TOF-MS/MS analysis. Furthermore, the cytotoxic potential of the biotransformed products was compared to their parent substrate (S) using MTT assay against 2 cancer cell lines; A549 and Caco-2. After 72 h. incubation, the BPs showed concentration-dependent cytotoxic activity against Caco-2 and A549 with IC_50_ equal to 0.32 and 0.78 mg/ml, respectively. Both BPs and S were safe on the normal human skin fibroblast (hFB) cell line. Molecular docking confirmed the high binding affinity of A7P towards phosphatidyl inositide 3-kinase delta (PI3K*δ*) and Cathepsin B protein (CatB) expressed in lung and colorectal cancer cells compared to quercetin suggesting higher anticancer activity. The present findings suggest that the application of biotransformation techniques using bacteria as *B. subtilis* can enhance the biological activity of flavonoids by generating bioactive metabolites.

**Supplementary Information:**

The online version contains supplementary material available at 10.1186/s13568-025-01860-2.

## Introduction

Flavonoids are a group of bioactive polyphenol compounds that widely exist in vegetables, fruits and medicinal herbs (Alice et al. 2018). Most natural flavonoids are found in their glycosidic forms with different sugar moieties. Glycosylation stabilizes the aglycones and enhances their water solubility, bioavailability, and bioactivity (You et al. 2010). The participation of dietary flavonoids in human health improvement has become an axiom. Moreover, flavonoids exert a wide range of biological activities like antifungal, antibacterial, antiviral, anticancer, antiaging, and anti-inflammatory activities (Wang et al. 2020). The production of bioactive flavonoids has been achieved through many biotechnological strategies including; microbial biotransformation, plant cell co-transformation, metabolic engineering, and enzyme engineering (Cao et al. 2014). Microbial biotransformation is a natural mechanism that exploits the enzyme system normally present in microorganisms. It uses the whole cells or isolates the active enzymes to enhance the efficiency of pharmacologically active compounds and perform synthetically challenging reactions (Kırcı et al. 2023).

Among the therapies that require new active and safe treatments are anticancer drugs. Cancer is a fatal ailment induced by irregular and indiscriminate cells. It is the second prime death cause all over the world (Aamna et al. 2022; Izuchukwu and Conradie 2023). The most common malignant tumor is lung cancer causing about one-fifth of cancer-related global deaths (Kang et al. 2020). Colorectal cancers are also considered one of the most serious types of cancers representing the third major cancer incidence with nearly 9.4% annual cancer cases mortality (Esghaei et al. 2018). Flavonoids have displayed their possession of various anticancer effects through different mechanisms. They amend reactive oxygen species scavenging enzyme activities, engage in arresting the cell cycle, induce apoptotic pathways, enhance autophagy, and repress cancer cell proliferation and invasiveness (Kopustinskiene et al. 2020). One of the ways by which flavonoids suppress cancer cell proliferation is by inhibiting PI3K involved in the PI3K/Akt/mTOR pathway which represents one of the key factors that control cell proliferation, metabolism, angiogenesis, cell cycle progression, apoptosis, and autophagy (Liu et al. 2018). Moreover, flavonoids’ inhibitive activity of CatB up-regulation interferes with cancer progression and regulation of apoptotic degenerative processes (Ramalho et al. 2015).

The current investigation aimed to explore the possibility of obtaining new bioactive compounds through the microbial biotransformation of quercetin and identify major biotransformed compounds using liquid chromatography-electrospray time-of-flight tandem mass spectrometry (LC-ESI-TOF-MS/MS). It also intended to evaluate and compare the anticancer activity of the parent compound and the biotransformed products. The anticancer activity was validated on two cancer cell lines: lung A549 cell line and colorectal Caco-2 cell line, and through in silico receptor-ligand docking modeling using PI3K*δ* and CatB proteins to impart insight into the level of the binding probability.

## Materials and methods

### Microbial strains

Four bacterial strains; *Staphylococcus aureus* ATCC 6538, *Escherichia coli* K12, *Bacillus subtilis* ATCC 23,857, and *Pseudomonas aeruginosa* PAO1 and two fungal strains; *Candida albicans* ATCC 10,231 and *Aspergillus niger* ATCC 32,656 were supplied by the Department of Immunology and Microbiology, faculty of pharmacy, Cairo university. The bacterial strains were cultivated on nutrient agar at 37 °C for 24 h., while the fungal strains were cultivated on Sabouraud dextrose agar at 30 °C for 48 h.

### Culture media and substrate

Mueller-Hinton broth for bacterial culture was purchased from Titan Biotech LTD, Rajasthan, India. Sabouraud Dextrose broth for fungal culture was purchased from Himedia leading BioSciences company, Mumbai, Maharashtra, India. Standard quercetin was purchased from E. Merck Co., Darmstadt, Germany.

### General biotransformation procedure

Biotransformation was executed using a standard two-stage protocol with some modifications (Cichewicz and Kouzi 1998). Stage I: Bacterial cultures were prepared by suspending the cells from agar slant in 50 ml of Mueller-Hinton broth at 250-ml Erlenmeyer flask and incubated for 24 h. on a shaker incubator at 180 rpm and 37 °C. Fungal cultures were prepared similarly using 50 ml of Sabouraud Dextrose broth but incubated in a shaker incubator at 180 rpm and 30 °C. Stage II: Cultures were initiated by inoculating 40 ml of fresh, sterile broth with 10 ml of stage I culture broth and incubated in a shaker for 24 h. Quercetin (25 mg dissolved in 2.5 ml DMSO) was added to each broth culture to produce final concentration of 0.5 mg/ml. Two flasks were prepared for each microbe; one was incubated in a shaker incubator and the other was incubated in a regular incubator (without shaking).

Fermentation was allowed for 24 h. Samples (0.5 ml) were aseptically withdrawn, extracted with ethyl acetate (0.5 ml), mixed for 2 min using a vortex, and centrifuged (13,000 rpm) for 5 min. For each sample, the upper layer was investigated for possible biotransformation using thin layer chromatography (TLC). Pre-coated silica TLC plates 60 F254 (20 × 20 cm, 0.25 mm thickness, Macherey Nagel, Germany) were used as stationary phase, while the mobile phase was chloroform: ethyl acetate: formic acid (60:40:0.01 v/v). Spots were examined in visible and UV light. The disappearance of the parent compound spot and the appearance of new spot/s with different Rf value/s were considered positive sign of biotransformation. Positive/substrate controls prepared from sterile broth were incubated with quercetin without microbes. Negative/culture controls were prepared of broth in which microorganisms were grown under identical conditions without quercetin addition. The flasks which showed positive signs of biotransformation were centrifuged (6000 rpm) for 5 min to separate the bacteria from the broth. Upon lyophilization of 25 ml of the broth, 308 mg dried biotramsformed products (BPs) were obtained. BPs were stored under refrigeration (4°C) until further testing.

### LC-ESI-TOF-MS/MS analysis of the biotransformed products

#### Sample preparation

A stock solution of BPs was prepared by dissolving 50 mg in 1 ml of a reconstitution solvent composed of water, methanol, and acetonitrile in a ratio of 2:1:1 v/v. Complete solubilization of the stock solution was achieved by subjecting it to vortex for 2 min and ultrasonication at 30 kHz for 10 min. The stock solution was then centrifuged for another 10 min at 10,000 rpm. An aliquot, 50 µl of stock solution was diluted to 1000 µl by reconstitution solvent to prepare a final concentration of 2.5 µg/µl, and 10 µl was used for injection in negative mode. A blank sample was also injected using 10 µl of reconstitution solvent.

#### Instruments and acquisition method

Separating small molecules was executed on an Exion LC system (AB Sciex, USA) linked to an autosampler system, a filter disks pre-column (3.0 mm × 0.5 μm, Phenomenex, USA), and an X select HSS T3 (2.1 × 150 mm, 2.5 μm) column (Waters Corporation, Milford, MA, USA) kept at 40 °C. The applied flow rate was 300 µl/min. The mobile phase was composed of 100% acetonitrile (A) and 5 mM ammonium formate in 1% methanol. The pH was adjusted to 3 using formic acid to form solution (B) for positive mode or adjusted to pH 8 using ammonium hydroxide to form solution (C) for negative mode.

The mass spectrometry (MS) was carried out on a Triple TOF 5600 + system provided with a Duo-Spray source working in the ESI mode (AB SCIEX, Concord, ON, Canada). The Triple TOF system was operated using an information-dependent acquisition (IDA) protocol, which was used to assemble full-scan MS and MS/MS information. Thereafter, the top 15 intense ions were chosen for MS/MS fragmentation spectra acquisition after each scan (Hegazy et al. 2021).

#### LC-MS data processing

Non-targeting, small molecule inclusive analysis of the sample was performed using MS-DIAL 3.70 open-source software (Tsugawa et al. 2015). GNP negative (2351 records) databases were utilized as reference databases. The MS-DIAL output was utilized to run on MasterView which was used for feature (peaks) extraction from total ion chromatogram based on standard criteria (Mohammed et al. 2021).

### Evaluation of the cytotoxicity of the biotransformed products

#### Cell lines, cell culture, and substrate preparation

Adeno-carcinomic human alveolar basal epithelial cells: A549 cell line and adeno-carcinomic human colorectal epithelial cells: Caco-2 cell line were purchased from the American Type Cell Culture (ATCC, USA), while normal human primary skin fibroblast: hFB cell line was purchased from Rio de Janeiro Cell Bank (BCRJ, Brazil). All cells were cultured in Dulbecco’s Modified Eagle (DMEM) high glucose medium which comprised 10% fetal bovine serum (FBS), 2 mM L-glutamine, and 1% antibiotic-antimycotic cocktail. All were purchased from Biowest (Nuaillé, France). Cells were kept in humidified air having 5% CO_2_ at sub-confluency at 37 °C. For sub-culturing step, monolayer cells were treated with trypsin/EDTA then harvested at 37 °C. Cells were used upon reaching 75% confluency. The substrate (S) was prepared by mixing 0.5 mg of quercetin with 21.5 mg of Mueller Hinton broth.

#### Cytotoxic assay (MTT)

The MTT colorimetric assay was conducted based on the original procedure proposed by Hansen et al. (Hansen et al. 1989). The MTT (3-[4,5-dimethylthiazole-2-yl]-2,5-diphenyltetrazolium bromide) was purchased from Merck KGaA (Darmstadt, Germany). It was utilized to evaluate the examined samples’ cytotoxicity. The assay is based on the ability of the living cells’ active mitochondrial dehydrogenase enzyme to cleave the yellow MTT tetrazolium rings forming insoluble dark blue formazan crystals. The crystals’ solubilization leads to the dark blue color formation directly proportional to the live cells’ number. In brief, cells (1 × 10^4^ cells/well) in serum-free media were seeded in a 96-well flat bottom microplate and treated with 20 µl of different concentrations of the examined samples for 72 h. in a humidified air with 5% CO_2_ at 37ºC. The samples’ final concentration range was 3.125 to 1000 µg/ml. At the end of the incubation period, media were discarded. MTT solution (40 µl) was added for each well and incubated for 4 hr. The MTT crystals solubilization was executed by adding acidified isopropanol 180 µl/ well. The plates were shaken at room temperature. The photometric absorbance was determined at 570 nm using a microplate ELISA reader (FLUOstar OPTIMA, BMG LABTECH GmbH, Ortenberg, Germany). Each concentration was repeated twice and the average was calculated. The formula used to calculate the cytotoxicity was as follows:

$$\begin{aligned}{\text{Percentage of relative viability}}=\\ \frac{{OD\,of\,the\,sample}}{{OD\,of\,the\,control}} \times 100\end{aligned}$$

#### Docking of quercetin and A7P to the binding sites of PI3K***δ*** and Cat B

The crystal structure of PI3K*δ* (PDB ID: 8BCY, PDB DOI: 10.2210/pdb8B4T/pdb) and CatB (PDB ID: 8B4T, PDB DOI: 10.2210/pdb8BCY/pdb) were downloaded from Protein Data Bank (https://www.rcsb.org). 8BCY consists of two chains: chain A (Phosphatidylinositol 4,5-bisphosphate 3-kinase catalytic subunit delta isoform, Gene Names: PIK3CD) and chain B (Phosphatidylinositol 3-kinase regulatory subunit alpha, Gene Names: PIK3R1) (Mazzucato et al. 2023). 8B4T consists of one chain (Cathepsin B, Gene Names: CTSB, CPSB) (Rubesova et al. 2023). All the molecular modeling studies were carried out by Molecular Operating Environment (MOE, 2019.0102) software. The proteins were firstly prepared by eliminating the crystallographic water molecules. Only the chain that is co-crystallized with the ligand was kept. The selected protein chain was refined to the root-mean-square (RMS) gradient of 0.001 kcal/mol /A^2^ and energy minimized to 10^− 5^ kcal/mol /A. The active sites of the target proteins were identified for docking study using pre-co-crystallized ligands; 9-[2-(3,4-dichlorophenyl) ethyl]-2-(3-hydroxyphenyl)-8-oxidanylidene-7~{H}-purine-6-carboxamide C_20_H_15_C_l2_N_5_O_3_ (DHOPC) for PI3K*δ* and (2S)-2-[[(2S)-2-[(4-chloranylphenoxy) carbonyl amino]-3-cyclohexyl-propanoyl] amino]-3-phenyl-propanoic acid C_25_H_29_ClN_2_O_5_ (PPA) for CatB. The grid co-ordinates of the interacting amino acids with the pre-co-crystallized ligands were 13.197 (X), -1.856 (Y), and 17.052 (Z) for 8BCY, and 12.335 (X), -0.512 (Y), and − 3.466 (Z) for 8B4T.

The 2D structures of the parent compound (quercetin) and the main biotransformed product (A7P) were drawn using ChemDraw professional 17.0 software. The structures were copied as smiles and pasted on MOE interface as 3D structures. The proposed compounds were protonated. Their energies were minimized using RMS gradient 0.1 Kcal/mol /A^2^ to obtain the most stable conformers. For each docking run, 10 poses were generated using the Triangle Matcher placement method and London dG scoring function. The docking results were visualized using Biovia Discovery Studio Visualizer 2024. The pose with ideal binding mode for each compound was used in portending the ligand–enzyme interactions at the active site. Analysis of the docking results was performed by comparing the interactions and docking scores of the docked compounds with that of the pre-co-crystallized ligand.

### Statistics

Data is expressed as mean ± SD. Non-linear regression analysis was used in calculating the dose-response curve with 5 parametric logistic curve equations using GraphPad Prism 10.2.2 (341) (San Diego, California USA, www.graphpad.com).

## Results

### LC-ESI-TOF-MS/MS analysis of the biotransformed products

**Fig. 1:**
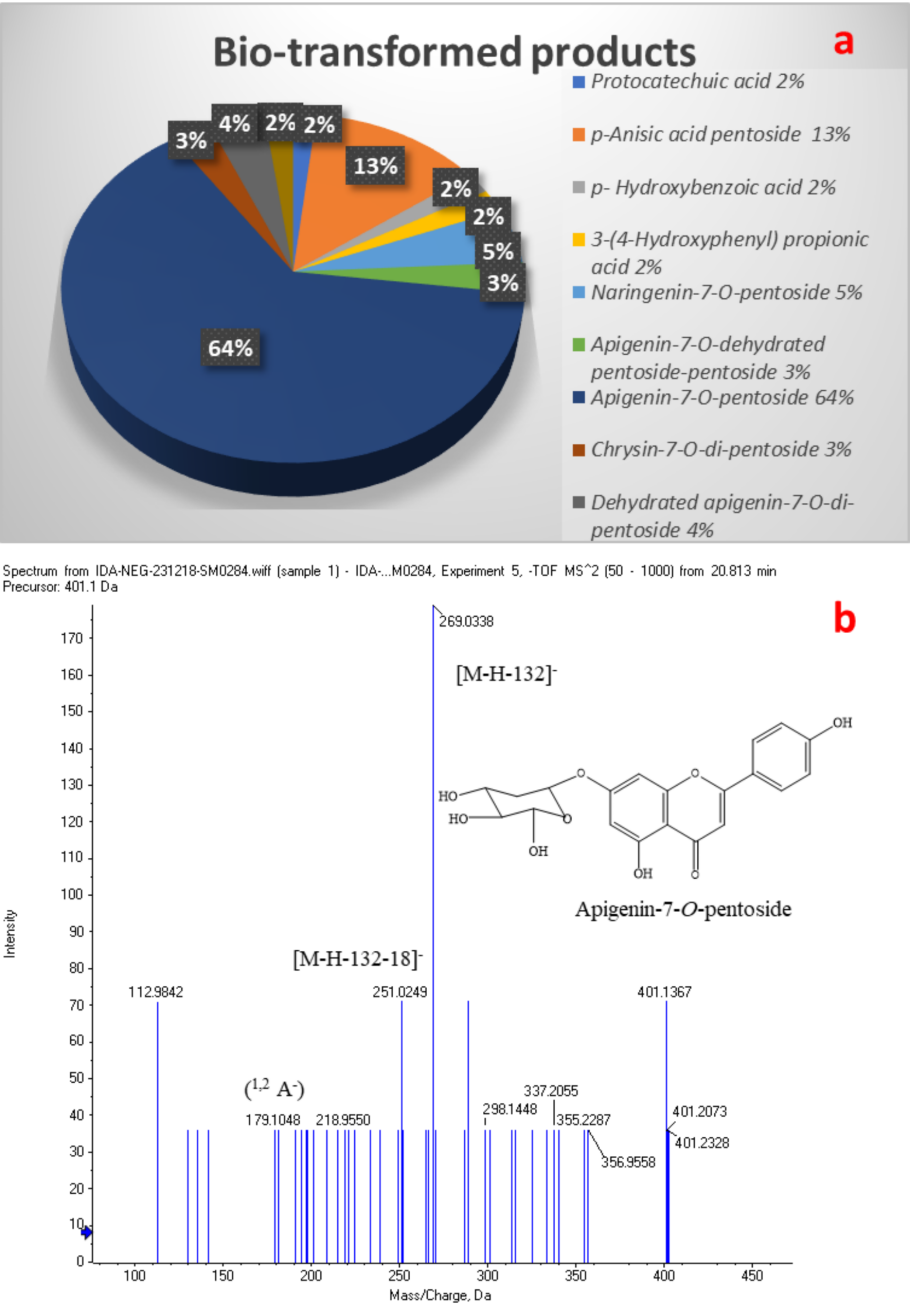
**a** The relative percentages of the biotransformed products detected in the broth after incubation of 24 h. **b** MS/MS spectrum of the main biotransformed product A7P (7)

In the present study, a preliminary screening of the collected samples revealed the ability of *B. subtilis* ATCC 23,857 under aerobic condition (with shaking) to biotransform quercetin which disappeared completely from the incubation media within 24 h. *B. subtilis* ATCC 23,857 under anaerobic conditions and the other microbial strains under both conditions did not show any positive signs of biotransformation. LC-ESI-TOF-MS/MS analysis of BPs in negative mode after 24 h. of incubation unveiled the characterization of 10 compounds which are reported in Table 1 and their relative percentages calculated from the peak area are displayed in Fig. 1a.

**Table 1:** Compounds identified in the biotransformed lyophilized broth in LC-ESI-TOF-MS/MS in negative mode

Comp. num.	Proposed compound (PubChem CID)	Molecular formula	Retention time Rt	Molecular ion (M-H)^−^	Mass fragmentation
1	Protocatechuic acid (72)	C_7_H_5_O_4_^−^	1.38	153.0193	**109**, 91, 81, 65
2	*p*-Anisic acid pentoside	C_13_H_15_O_7_^−^	1.45	283.0676	**151**, 108
3	*p*-Hydroxybenzoic acid (135)	C_7_H_5_O_3_^−^	9.05	137.0250	119,109,**93**, 65
4	3-(4-Hydroxyphenyl) propionic acid (10394)	C_9_H_9_O_3_^−^	9.13	165.0557	137,**121**, 79, 60
5	Naringenin-7-*O*-pentoside	C_20_H_19_O_9_^−^	17.52	403.1310	403, **271**, 253
6	Apigenin-7-*O*- dehydrated pentoside-pentoside	C_25_H_23_O_12_^−^	18.63	515.1078	515, 469, 383, 269, 175, **113**
7	Apigenin-7-*O*-pentoside	C_20_H_17_O_9_^−^	20.81	401.1367	401, **269**, 251, 179
8	Chrysin-7-*O*-di-pentoside	C_25_H_25_O_12_^−^	22.57	517.2099	517, 471, **385**, 353, 253, 177
9	dehydrated apigenin-7-*O*-di-pentoside	C_25_H_23_O_12_^−^	22.62	515.2154	515, 469, **383**, 353, 251
10	Taxifolin-7,4’-*O*-dimethyl-3-*O*-deoxyhexoside	C_23_H_25_O_11_^−^	23.01	477.2877	477, **331**, 313, 205

Base peaks are bolded

Compounds **1**, **3**, and **4** having [M-H]^−^ at *m/z* 153.0193, 137.025, and 165.0557 exhibit base peaks at *m/z* 109, 93, and 121, respectively. It was attributed to the loss of CO_2_. They were identified as protocatechuic acid (Fig. S1), *p*-hydroxy benzoic acid (Fig. S3), and 3-(4-hydroxyphenyl) propionic acid (Fig. S4), respectively. Compound **2** with [M-H]^−^ at *m/z* 283.0676 shows loss of pentose moiety to give a base peak at *m/z* 151. Further loss of CO_2_ gave a fragment ion at *m/z* 108. It was characterized as *p*-anisic acid pentoside (Fig. S2). Compound **5** having [M-H]^−^ at *m/z* 403.1310 shows a loss of pentose moiety giving a base peak at *m/z* 271 (aglycone). Further loss of water produced the fragment ion at *m/z* 253. It was identified as naringenin-7-*O*-pentoside (Fig. S5). Compound **6** shows [M-H]^−^ at *m/z* 515.1078. The loss of pentose moiety resulted in a fragment ion at *m/z* 383. The aglycone ion (Y_0_) at *m/z* 269 [M-H-132-114]^−^ was produced upon loss of dehydrated pentose. It was assigned as Apigenin-7-*O*-dehydrated pentoside-pentoside (Fig. S6). Compound **7** with [M-H]^−^ at *m/z* 401.1367 exhibits a base peak at *m/z* 269 (aglycone) which was attributed to the loss of pentose moiety. Two fragment ions are observed at *m/z* 251 [aglycone-18]^−^ and *m/z* 179 (^1,2^ A^−^). It was identified as apigenin-7-*O*-pentoside (A7P) (Fig. 1b). Compound **8** having [M-H]^−^ at *m/z* 517.2099 shows two consecutive losses of pentose moieties to give a base peak at *m/z* 385 and a fragment ion at *m/z* 253 (chrysin aglycone). Further fragmentation gave a product ion at *m/z* 177 [aglycone-44-18-14]^−^. It was identified as chrysin-7-*O*-di-pentoside (Fig. S7). Compound **9** having [M-H]^−^ at *m/z* 515.2154 exhibits two consecutive losses of pentose moieties yielding a base peak at *m/z* 383 and a fragment ion at *m/z* 251. It was identified as dehydrated apigenin-7-*O*-di-pentoside (Fig. S8). Compound **10** with [M-H]^−^ at *m/z* 477.2877 shows loss of deoxyhexose to produce a base peak at *m/z* 331 (aglycone). A fragment ion was observed at *m/z* 313 [M-H-146-18]^−^. It was assigned as taxifolin-7,4'-*O*-dimethyl-3-*O*-deoxyhexoside (Fig. S9).

### Microbial enzymatic reactions involved in quercetin biotransformation

**Fig. 2:**
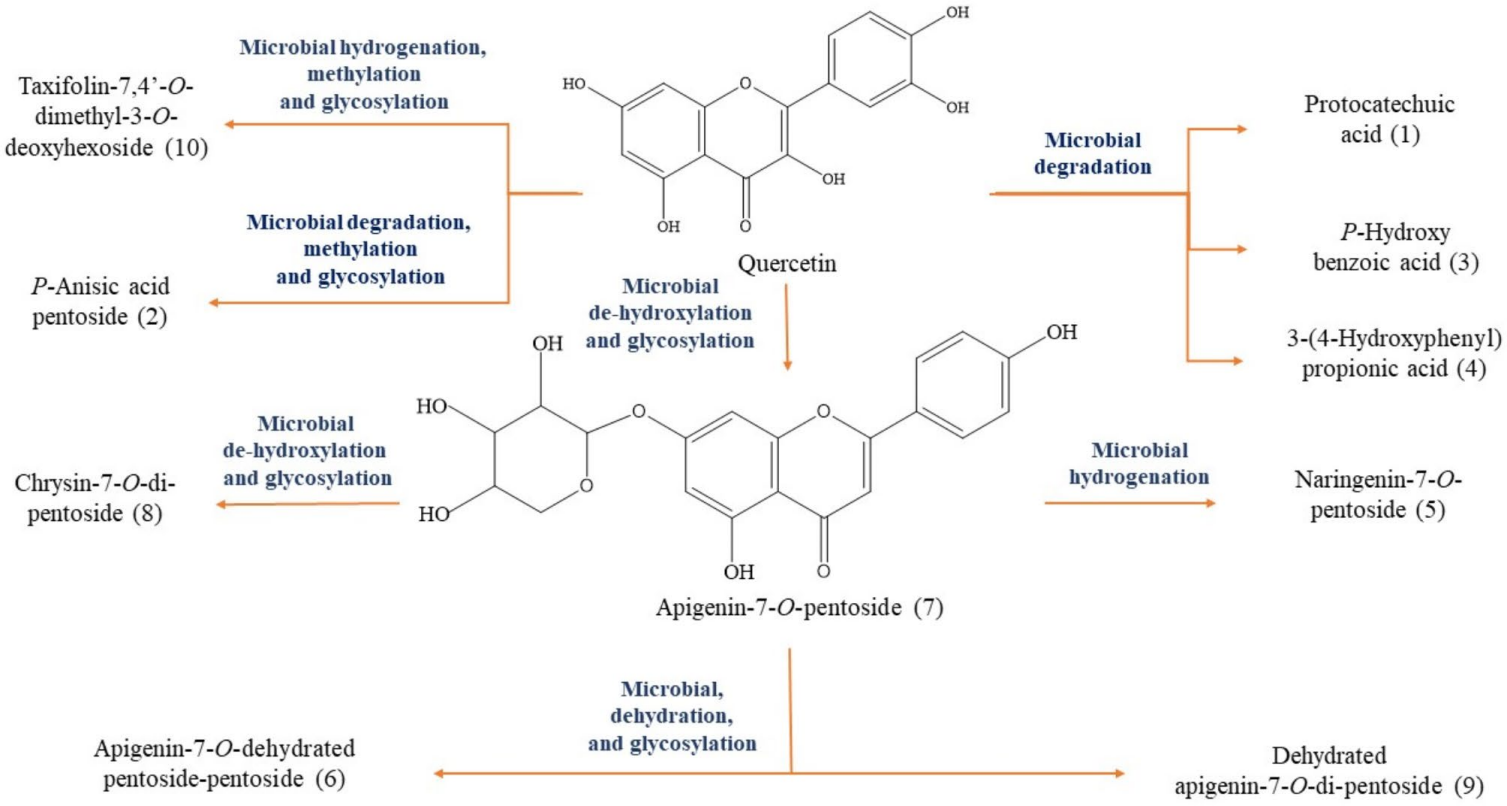
The biotransformed metabolites of quercetin by *B. subtilis* ATCC 23,857 and the proposed microbial enzymatic reactions

The biotransformation of quercetin employing *B. subtilis* ATCC 23,857 led to seven products. The major was A7P (7), constituting about 64% of the total converted compounds. Microbial dehydroxylation at 3 and 3'-positions produced apigenin which upon further glycosylation at 7-position gave the pentoside glycoside of apigenin. A7P underwent four more bioconversions; double bond reduction at 2,3-position giving naringenin-7-*O*-pentoside (5), dehydroxylation at 4'-position followed by glycosylation producing chrysin-7-*O*-di-pentoside (8), and dehydration followed by glycosylation resulting in apigenin-7-*O*-dehydrated pentoside-pentoside (6) and dehydrated apigenin-*7-O-*di-pentoside (9). Microbial hydrogenation at 1,2-position followed by methylation at 7 and 4' positions gave dimethylated taxifolin. Further glycosylation at 3-position resulted in taxifolin-7,4'-*O*-dimethyl-3-*O*-deoxyhexoside (10). Microbial degradation resulted in 3 products namely; protocatechuic acid (1), *p*-hydroxybenzoic acid (3), 3-(4-hydroxyphenyl) propionic acid (4). The second one was methylated and glycosylated to produce *p*-anisic acid pentoside (2). The biotransformed metabolites of quercetin by *B. subtilis* ATCC 23,857 and their proposed microbial enzymatic reactions are illustrated in Fig. 2.

### Evaluation of the cytotoxicity of the biotransformed products

**Fig. 3:**
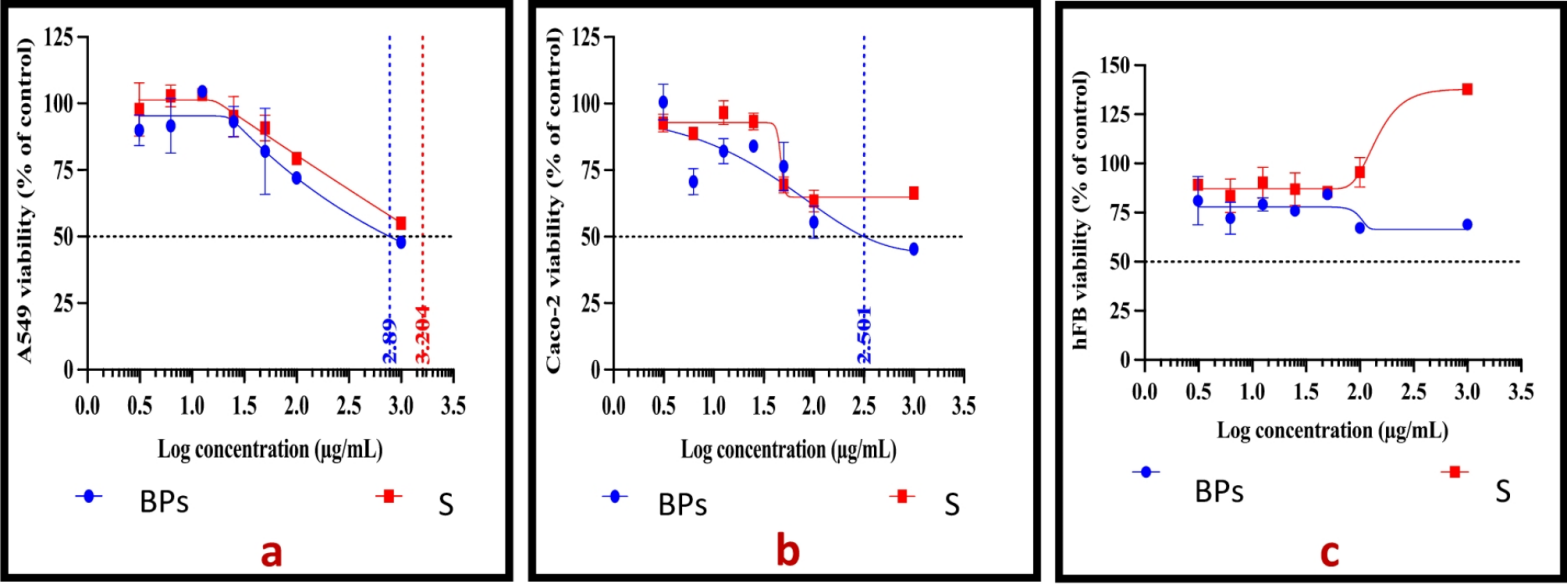
The dose-response curves of BPs and S against A549 **a**, Caco-2 **b**, and hFB **c** cell lines after 72 h

Using MTT assay, the cytotoxicity potential of BPs and S (2.33% quercetin in Mueller Hinton broth) were evaluated after 72 h. on two cancer and a normal cell line. The dose-response curves are shown in Fig. 3. Graph Pad Prism Software was used to calculate IC_50_ values. The results show that both BPs and S exhibited cytotoxic activity on the A549 cell line with IC_50_ values of 0.78 and 1.58 mg/ml, respectively. When Caco-2 cells were treated with BPs, there was a concentration-dependent cytotoxic effect with IC_50_ values of 0.32 mg/ml. On the other hand, sample S showed about 35% toxicity on the Caco-2 cell line. On treating the hFB cell line with the different concentrations of both samples, no significant decrease in cell viability was observed with BPs indicating its safety. Moreover, the cell viability was increased with S reflecting proliferative effects and safety on normal hFB cell line.

### Docking of quercetin and A7P to the binding sites of PI3K***δ*** and Cat B

**Fig. 4:**
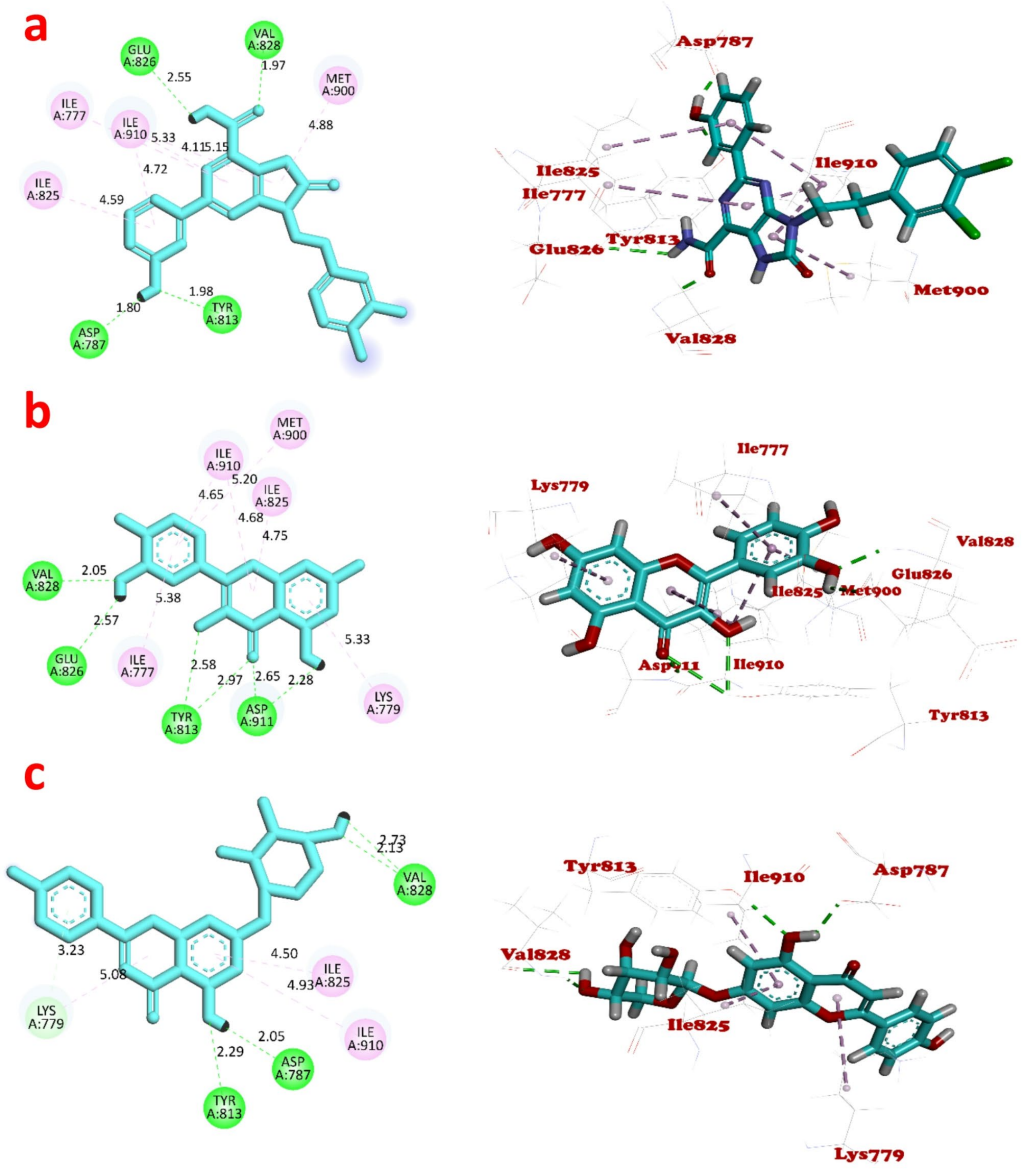
The 2D (two-dimensional) and 3D (three-dimensional) views of **a** DHOPC, **b** quercetin, **c** A7P interactions with Human PI3Kδ protein using Discovery studio. The bond lengths are illustrated in the 2D views. The bond interactions; the hydrogen, pi-alkyl, and pi-donor hydrogen are shown as green, pink, and light blue broken lines, respectively. The ligand backbones are colored blue

**Fig. 5:**
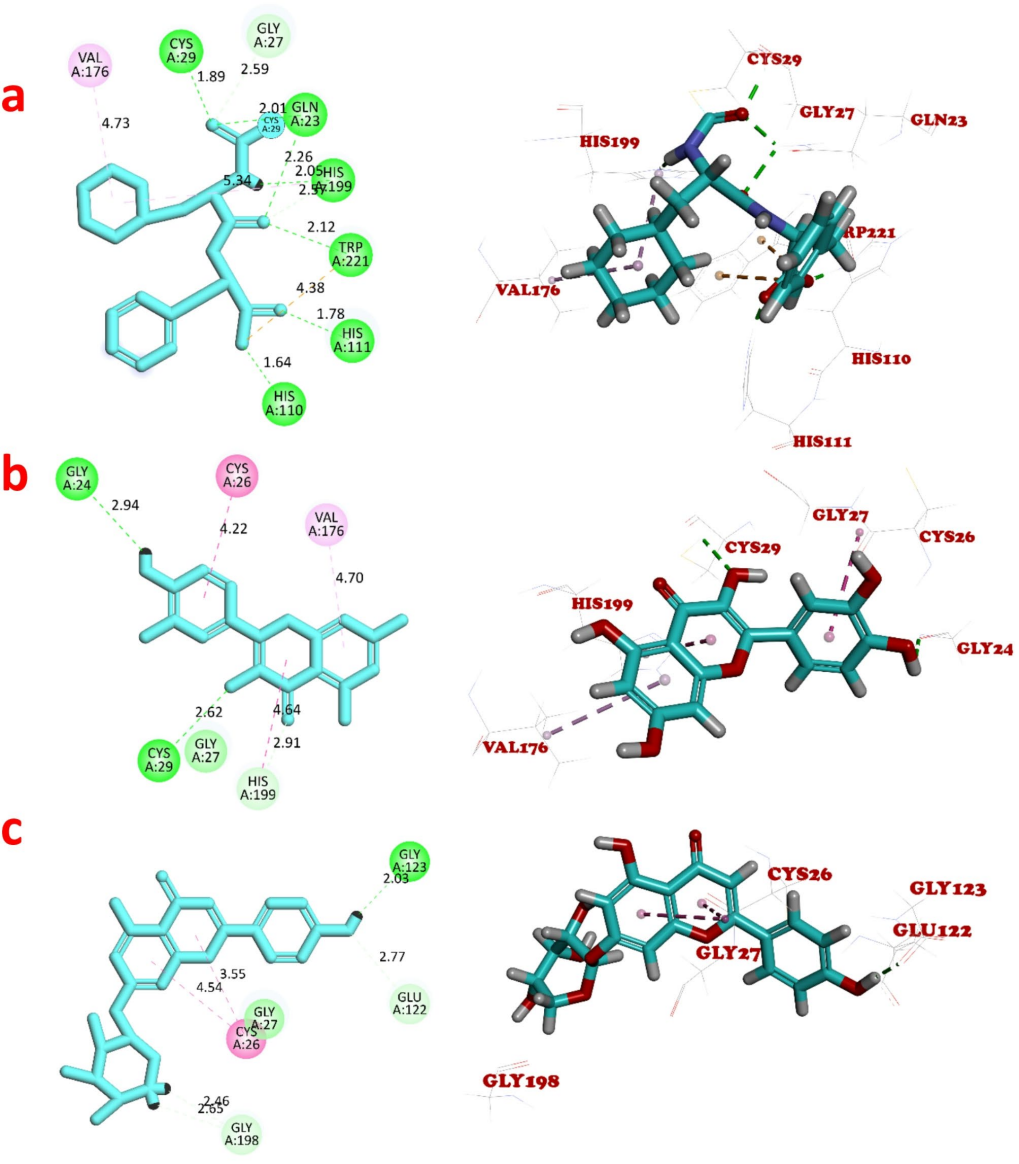
The 2D and 3D views of **a** PPA, **b** quercetin, **c** A7P interactions with Human cathepsin B using Discovery studio. The bond lengths are illustrated in the 2D views. The bond interactions; the hydrogen, carbon hydrogen, pi-alkyl, amide-pi stacked, van der waals, and pi-anion are shown as green, light blue, pink, purple, light green, and orange broken lines, respectively. The ligand backbones are colored blue

Molecular docking was held using two proteins. Human PI3K*δ* (PDB ID: 8BCY) is a transferase enzyme consisting of two protein chains; chain A has 1018 amino acids and chain B has 169 amino acids (Mazzucato et al. 2023). Human CatB (PDB ID: 8B4T) is a cysteine protease enzyme consisting of one chain of 255 amino acids (Rubesova et al. 2023). The binding energy (kcal/mol) of the tested compounds, type of interacting residues, and the interacting groups in the binding site of PI3K*δ* and CatB are illustrated in Table 2. The 2D ligand interaction diagrams and the corresponding 3D representation of the predicted binding mode of the tested compounds with the active site of PI3K*δ* protein and CatB are shown in Figs. 4 and  5, respectively. At first, the docking setup was validated using self-docking of the co-crystallized ligands DHOPC and PPA in the proximity of the binding sites of the PI3K*δ* and CatB, respectively. DHOPC shows 4 conventional hydrogen bonds and 4 pi-alkyl interactions with the binding sites of PI3K*δ* (Fig. 4a). PPA docking with CatB binding sites exhibits 7 conventional hydrogen bonds with 6 amino acids, 2 carbon hydrogen bonds, 2 pi-alkyl hydrophobic interactions, and a pi-anion electrostatic interaction (Fig. 5a).

**Table 2:** Molecular docking analysis of quercetin, A7P, and the co-crystallized ligands with PI3K*δ* and CatB binding sites

Protein (PDB ID)	Ligand	Binding score (kcal/mol)	Hydrogen bond interacting residues			Hydrophobic interacting residues	
			AA	IG	BL	AA	BL
Human PI3K delta (8BCY)	DHOPC	− 7.6042	ASP 787	OH (H29)	1.80	MET 900	4.88
			TYR 813	OH (O29)	1.97	ILE 777	5.33
			VAL 828	C=O (O3)	1.98	ILE 825	4.59
			GLU 826	NH (H11)	2.55	ILE 910	4.72
	Quercetin	− 6.1778	ASP 911	C=O (O4)	2.65	ILE 910	4.65
			ASP 911	OH (H7)	2.28	ILE 825	4.75
			TYR 813	C=O (O4)	2.97	ILE 777	5.38
			TYR 813	OH (O3)	2.58	MET 900	5.20
			VAL 828	OH (O3’)	2.05	LYS 779	5.33
			GLU 826	OH (H3’)	2.57		
	A7P	− 7.7770	VAL 828	OH (O4”)	2.13	LYS 779	5.08
			VAL 828	OH (H4”)	2.73	ILE 910	4.93
			TYR 813	OH (O5)	2.29	ILE 825	4.50
			ASP 787	OH (H5)	2.05		
			LYS 779	Pi-Orbitals	3.23		
Human CatB (8B4T)	PPA	− 7.2056	CYS 29	C=O (O2)	1.89	VAL 176	4.73
			GLN 23	C=O (O2)	2.01	HIS 199	5.34
			GLN 23	C=O (O3)	2.26		
			HIS 199	NH (H3)	2.05		
			TRP 221	C=O (O3)	2.12		
			HIS 111	C=O (O4)	1.78		
			HIS 110	OH (O1)	1.64		
			GLY 27	C=O (O2)	2.59		
			HIS 199	C=O (O3)	2.57		
	Quercetin	− 5.9811	CYS 29	OH (O4’)	2.62	HIS 199	4.64
			GLY 24	OH (O3)	2.94	CYS 26	4.22
			HIS 199	C=O (O4)	2.91	VAL 176	4.70
	A7P	− 6.9158	GLY 123	OH (H4’)	2.03	CYS 26	3.55
			GLU 122	OH (H4”)	2.77	CYS 26	4.54
			GLY 198	CH (H5”)	2.46		
			GLY 198	CH (H5”)	2.65		

AA, amino acids; IG, interacting groups; BL, bond length; VAL, Valine; GLU, Glutamic acid; TYR, Tyrosine; ILE, Isoleucine; MET, Methionine; LYS, Lysine; CYS, Cysteine; GLN, Glutamine; HIS, Histadine; TRP, Tryptophan; GLY, Glycine; ASP, Aspartic acid.

Molecular docking of quercetin with the binding sites of PI3K*δ* shows 6 conventional hydrogen bonds with 4 amino acids residues. The remaining amino acids exhibit pi-alkyl hydrophobic interactions (Fig. 4b). When quercetin was docked with the binding sites of CatB, only 2 conventional hydrogen bonds and a carbon hydrogen bond were observed. Quercetin also exhibits 3 hydrophobic interactions; pi-alkyl with Val 176, pi-pi T-shaped with His 199, and amide-pi Stacked with Cys 26 (Fig. 5b). Molecular docking of A7P with PI3K*δ* binding sites demonstrates 4 conventional hydrogen bonds with 3 different amino acids, a pi-donor hydrogen bond with Lys 779, and 3 pi-alkyl interactions (Fig. 4c). Docking of A7p with CatB binding sites shows a conventional hydrogen bond with Gly 123 and 3 carbon hydrogen bonds. Two amide-pi Stacked hydrophobic interaction of A7P was noticed with Cys 26 (Fig. 5c). Tyr 813, Val 828, Ile 825, and Ile 910 were amino acid residues accountable for the binding of all ligands to the PI3K*δ*. No common amino acid binding residues were observed in all ligands of CatB. The tested ligands generally show better binding affinity to the PI3K*δ*’s active sites than the CatB active sites. The main biotransformed product A7P shows a binding score value (− 7.7770 kcal/mol) for PI3K*δ* that is lower than that of the co-crystallized ligand (DHOPC) (− 7.6042 kcal/mol) and that of the parent substrate (− 6.1778 kcal/mol). A7P also shows a binding score (− 6.9158 kcal/mol) for CatB which is higher but comparable to that of the co-crystalized ligand (PPA) (-7.2056 kcal/mol) and lower than that of quercetin (− 5.9811 kcal/mol). These results suggest better anticancer activity of A7P than quercetin.

## Discussion

The present study explored the ability of *B. subtlis* ATCC 23,857 to stimulate metabolic reactions that led to the bioconversion of quercetin; an important flavonoid belonging to the flavonol class and widely exist in many vegetables and fruits (Magar and Sohng 2020). Ten biotransformed products were detected using LC-ESI-TOF-MS/MS analytical method. The main biotransformed product was apigenin-7-*O*-pentoside (7) (A7P); a new bioconverted product of quercetin. Six other phenolic compounds were detected for the first time as biotransformed quercetin products. Additionally, this work highlighted the possible enzymatic reactions that may have been involved in the biotransformation process. Quercetin bioconversion was previously investigated using many filamentous fungi, for example *Beauveria bassiana* ATCC 7159 (Araújo et al. 2013) and *Gliocladium deliquescens* NRRL 1086 (XU et al. 2017). In addition, many bacterial strains succeeded in quercetin bioconversion, such as *Bacillus cereus* (Rao and Weisner 1981) and *Bacillus* sp. CSQ 10 (Kang et al. 2022). The metabolic engineering of *B. subtilis* 168 strain was reported to biotransform quercetin to isoquercetin (Niu et al. 2024). Biotransformation of quercetin was also achieved using plant suspension cultures which can be considered as an important tool of glycosidic production (Popova et al. 2021).

Four main microbial enzymatic reactions were described in our study namely; dehydroxylation, glycosylation, hydrogenation, and methylation. The microbial reactions involved in the production of the main biotransformed product, A7P, were the dehydroxylation and the glycosylation. Flavonoid dehydroxylation was previously reported in the conversion of rutin (quercetin 3-*O*-rutinoside) to kaempferol 3-*O*-rutinoside (Lin et al. 2014). Biotransformation of hesperidin to naringenin involved 3'-dehydroxylation which was achieved using *Aspergillus niger* ATCC 10,549. In the same study, hesperidin was converted to pinocembrin by *Alternaria alternata* NRRL 20,593 in which 3' and 4' dehydroxylation were involved (Kırcı et al. 2023). Microbial glycosylation is considered one of the most popular modification strategies for regulating the solubility, stability, bioavailability, and bioactivity of phytochemical drugs (Wang et al. 2020). In addition, glycosylation has been involved in obtaining many novel metabolites that are absent in natural sources (Magar and Sohng 2020). Many studies reported the microbial biotransformation of quercetin to isoquercetin (quercetin-3-*O*-*β*-D-glucopyranoside) which is more water soluble and has many biological activities, such as anticarcinogenic, antioxidant, cardioprotective, anti-inflammatory, antidiabetic, and anti-allergic activities (Rao and Weisner 1981; Xu et al. 2017; Popova et al. 2021; Kang et al. 2022). In the current study, the glycosylation of the major compound and most bioconverted products was through the addition of pentose sugar. This can be attributed to the pentose phosphate pathway normally present in *B. subtilis* which converts hexose to pentose (Blencke et al. 2003). Flavonoid-*O*-pentosides were previously synthesized using *E. coli* through the bioengineering of nucleotide sugar pathways and glycosyltransferase (Pandey et al. 2013; Han et al. 2014). In nature, glycosyltransferases play an important role in most glycosylation reactions by assisting the transfer of sugar residue from an activated sugar donor, mostly nucleotide sugars, to various metabolites. The highest percentage of nucleotide sugars are the uridine diphosphate (UDP) sugars, predominantly UDP-glucose, UDP-glucuronate, UDP-rhamnose, UDP-arabinose, and UDP-xylose. These sugars consequently perform the large class of glycosyltransferases, recently been used for the glycosides biosynthesis in microorganisms (Bruyn et al. 2015).

Microbial hydrogenation of flavonoids was previously reported. An obligatory anaerobe of the human intestinal tract, *Eubacterium ramulus*, successfully biotransformed quercetin to taxifolin (Braune et al. 2001). The human intestinal microbiota converted apigenin-7-glucoside to naringenin upon incubation with fecal suspension (Hanske et al. 2009). Methylation is a significant modification strategy that has long been employed and has offered many bioactive derivatives (Magar and Sohng 2020). Methoxy ring-B metabolites were obtained from quercetin biotransformation with *Streptomyces griseus* ATCC 13,273 (Hosny et al. 2001) and *Beauveria bassiana* ATCC 7159 (Costa et al. 2008).

Three degradation products of quercetin recognized in our study are protocatechuic acid, *p*-hydroxybenzoic acid, and 3-(4-hydroxyphenyl) propionic acid. Decomposition of quercetin to protocatechuic acid is a common degradation pathway, which indicates the *B. subtilis* ability to split heterocyclic C-ring of quercetin. This ability can be attributed to quercetin 2,3-dioxygenase of *B. subtilis* QueD; the only known prokaryotic organism that produces this enzyme. Quercetin 2,3-dioxygenase converted quercetin to 2-protocatechuoyl phloroglucinol carboxylic acid which upon further hydrolysis produced protocatechuic acid (Schaab et al. 2006). Another study attributed the quercetin catabolism in *B. subtilis* 168 to a quercetin dioxygenase gene, seven oxidoreductase genes, and three ring-cleavage dioxygenase genes (Niu et al. 2024). Phloroglucinol is another intermediate in several flavonoid degradation pathways, but its degradation occurs instantly, which can explain why it could not be detected in our study (Braune et al. 2001). The obtainment of 3-(4-hydroxyphenyl) propionic acid and *p*-hydroxybenzoic acid from quercetin decomposition by gut microbiota was reported (Version and Please 2021). Five gut microbiotas; *Olsenella scatoligenes*,* Flavonifractor plautii*, *Eubacterium eligens*, *Bacteroides eggerthii*, and *Bacillus glycinifermentans* exhibited their ability to biotransform quercetin into different bioactive metabolites as trihydroxy benzoic acid, dihydroxybenzoic acid, and dihydroxy phenylacetic acid (Sankaranarayanan et al. 2021).

The present study examined and compared the cytotoxic activities of the BPs and S using in vitro MTT assay. A549 and Caco-2 cells were selected as models of tumor cell lines. BPs showed better cytotoxic activity than S towards both cell lines. The viability of normal hFB cells remained unaffected on applying BPs and increased with S indicating their safety. According to many studies, flavonoids showed a broad range of anticancer activities including modulation of ROS-scavenging enzyme activities, contribution in arresting the cell cycle, autophagy induction, and apoptosis enhancement. In addition, flavonoids, as pro-oxidants factors, could repress cancer cells proliferation by inhibiting the epidermal growth factor receptor/mitogen activated protein kinase, phosphatidyl inositide 3-kinases, protein kinase B, CatB, and nuclear factor kappa-light-chain-enhancer of activated B cells (Vidal-albalat and González 2016; Kopustinskiene et al. 2020). CatB is a group of lysosomal-encapsulated cellular cysteine proteases, their up-regulation represents a key factor in cancer progression and the degenerative processes regulation. Consequently, cathepsins have been considered efficient cancer biomarkers and specific targets in drug design (Saroha et al. 2022). The inhibition of the PI3K pathway was among the mechanisms involved in the anticancer activities of epigallocatechin gallate, quercetin, and apigenin in the T47D and HFF cells, the human hepatoma cell line, and the T24 cell line, respectively (Kopustinskiene et al. 2020). Myricetin and quercetin have been deemed among the promising inhibitors of human CatB (Ramalho et al. 2015).

Molecular docking is one of the significant in silico techniques, which can portend the interaction mode between a target protein and a small ligand. Binding energy reflects the compound’s binding strength and its affinity degree to the target protein pocket. The lower the binding energy, the more preferable the compound is as a possible drug candidate (Iheagwam et al. 2019). In the current study, the parent compounds (quercetin) and its main biotransformed product (A7P) were docked into the binding sites of PI3K*δ* and CatB to compare their binding affinities which reflect their anticancer activities. Hydrogen and various hydrophobic stabilizing interactions were observed between the ligands and amino acid residues at the protein active sites. Although quercetin constructed a higher number of conventional hydrogen bonds than A7P, the A7P binding energy score was lower than that of quercetin, indicating higher anticancer activity of A7P. The docking study revealed the superiority of the main biotransformed product A7P binding affinity over the co-crystalized ligand of PI3K*δ* and a comparable binding affinity to the co-crystalized ligand of CatB.

Although quercetin biotransformation has been extensively studied, it is the first time, to the best of authors’ knowledge, to produce flavonoid pentoside glycosides from quercetin using *B. subtilis* ATCC 23,857. On that account, further investigation should be directed to microbial biotransformation of biologically active phytochemicals for the sake of producing more active drugs. However, further studies are needed to confirm our results on other cancer cell lines. Dynamics simulation and molecular mechanics energies techniques can be implemented to further verify docking results. In addition, in vitro and in vivo examination can be executed for further validation of the cytotoxic mechanisms and the physiological application of these results.

## Electronic supplementary material

Below is the link to the electronic supplementary material.

Supplementary Material 1

## Data Availability

The authors declare that the data supporting the findings of this study are available within the paper. Should any raw data files be needed in another format they are available from the corresponding author upon reasonable request.

## Abbreviations

BPs: Biotransformed products
TLC: Thin layer chromatography
LC-ESI-TOF-MS/MS: Liquid chromatography electrospray time-of-flight tandem mass spectrometry
DMEM: Dulbecco’s Modified Eagle
FBS: Fetal bovine serum
A7P: Apigenin-7-O-pentoside
S: Substrate
MTT: (3-[4,5-dimethylthiazole-2-yl]-2,5-diphenyltetrazolium bromide) assay
PDB: Protein data base
PI3Kδ: Phosphatidyl inositide 3-kinase delta
and CatB: Cathepsin B protein
DHOPC: 9-[2-(3 4-dichlorophenyl) ethyl]-2-(3-hydroxyphenyl)-8-oxidanylidene-7~{H}-purine-6-carboxamide
PPA: (2 S)-2-[[(2 S)-2-[(4-chloranylphenoxy) carbonyl amino]-3-cyclohexyl-propanoyl] amino]-3-phenyl-propanoic acid
OD: Optical density
UDP: Uridine diphosphate
