# Supplementary material for: Biotransformation of quercetin by Bacillus subtilis and anticancer activity evaluation: in vitro and in Silico
Source: AMB Express. 2025 Apr 2;15:58. doi: 10.1186/s13568-025-01860-2 (PMC11965081; doi:10.1186/s13568-025-01860-2)
Supplement: Supplementary file 1 — Supplementary Material 1 [file 13568_2025_1860_MOESM1_ESM.docx]

**Supplementary material**

**Biotransformation of quercetin by *Bacillus subtilis* and anticancer activity evaluation: *in vitro* and *in silico***

**Salsabeel N. El Gendy^1^, Amira K. Elmotayam^1^, Reham Samir^2^, Marwa I. Ezzat^1*^, Mahmoud T. Abo-Elfadl^3,4^, Aly M. EL Sayed^1^**

^1^ Department of Pharmacognosy, Faculty of Pharmacy, Cairo University, Kasr El-Ainy Street, Cairo 11562, Egypt.

^2^ Department of Microbiology and Immunology, Faculty of Pharmacy, Cairo University, Kasr El-Ainy Street, Cairo 11562, Egypt.

^3^ Biochemistry Department, Biotechnology Research Institute, National Research Centre, Dokki, Cairo 12622, Egypt

^4^ Cancer Biology and Genetics Laboratory, Centre of Excellence for Advanced Sciences, National Research Centre, Cairo 12622, Egypt.

^*^ Correspondence:

Marwa I Ezzat,

Department of Pharmacognosy, Faculty of Pharmacy, Cairo University, Kasr El-Ainy Street, Cairo 11562, Egypt.

Tel.: +201009395028.

[M-H]^-^

[M-H- CO_2_] ^-^

**Fig. S1.** MS/MS spectrum of protocatechuic acid (1)

[M-H]^-^

[M-H-132]^-^

[M-H- CO_2_] ^-^

**Fig. S2.** MS/MS spectrum of *p-*Anisic acid pentoside (2)

[M-H]^-^

[M-H- CO_2_] ^-^

**Fig. S3.** MS/MS spectrum of *p*-hydroxy benzoic acid (3)

[M-H-28]^-^

[M-H- CO_2_] ^-^

**Fig. S4.** MS/MS spectrum of 3-(4-hydroxyphenyl) propionic acid (4)

[M-H]^-^

[M-H-132]^-^

[M-H-132-18]^-^

**Fig. S5.** MS/MS spectrum of naringenin-7-*O*- pentoside (5)

Y0

[M-H-132-114]^-^

[M-H-132]^-^

[M-H-46]^-^

**Fig. S6.** MS/MS spectrum of apigenin-7-*O*-dehydrated pentoside-pentoside (6)

[M-H]^-^

[M-H-132]^-^

Y0

[M-H-132-132]^-^

**Fig. S7.** MS/MS spectrum of chrysin-7-*O-*di-pentoside (8)

[M-H]^-^

[M-H-132]^-^

[M-H-132-132]^-^

**Fig. S8.** MS/MS spectrum of dehydrated apigenin-7-*O*-di-pentoside (9)

[M-H]^-^

[M-H-146]^-^

[M-H-146-18]^-^

**Fig. S9.** MS/MS spectrum of taxifolin-7,4’-O-dimethyl-3-O-deoxyhexoside (10)
